# Humanin P3S, haplogroup N1b and the risk of Alzheimer's disease

**DOI:** 10.1111/acel.14207

**Published:** 2024-05-17

**Authors:** Ian Stewart Logan

**Keywords:** Alzheimer's disease, Ashkenazi, Humanin, mitochondrial DNA, mitochondrial haplogroup

## Abstract

A commentary of the paper ‘Humanin variant P3S is associated with longevity in APOE4 carriers and resists APOE4‐induced brain pathology’ that appeared recently in Aging Cell. The possible association of a mitochondrial haplogroup with a disease is frequently discussed. The Humanin peptide encoded by the mtDNA has been shown to play an important regulatory role in cell metabolism. There are variants of Humanin caused by different mutations and it is known that the potent form of Humanin, termed S14G, is found naturally in the people of haplogroup U6a7a1a because they have the mutation m.A2672G; however it has not been shown that having this mutation is indeed beneficial. In their paper, the authors suggest that the mitochondrial DNA mutation, m.C2639T, may be beneficial in people who are in haplogroup N1b and also carry APOE4. The mutation changes the common form of Humanin to Humanin P3S. In the study, the researchers looked at a group of Ashkenazi women who were over the age of 95, and found that a higher proportion of them carried APOE4, suggesting that Humanin P3S protected them against the adverse effects of APOE4. A study in a mouse model supported this finding by showing treatment with Humanin P3S reduced APOE4‐induced brain pathology. In the world population, there are about 500,000 Ashkenazi in haplogroup N1b, predominantly in the subgroup N1b1b1; and there are about 9.5 million non‐Ashkenazi people with the mutation m.C2639T and are therefore also in haplogroup N1b and have Humanin P3S. However, the researchers have yet to show Humanin P3S is of benefit in non‐Ashkenazi people. This paper raises the possibility of a therapeutic use of Humanin P3S in the treatment of Alzheimer's disease.

## Graphical abstract

A commentary on the recent paper which associated the Humanin variant P3S with longevity in aged Ashkenazi women who carried APOE4. There are about 10 million people in the world who have the Humanin variant P3S, of which only about 500,000 are Ashkenazi. So does everyone with Humanin variant P3S have a lowered risk of developing Alzheimer's disease.

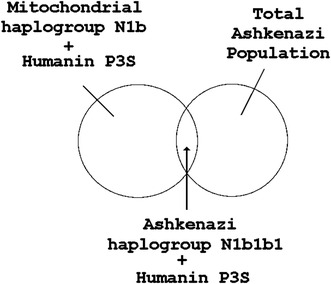

The role of mtDNA mutations and mtDNA haplogroups (which are a group of mtDNA lineages defined by a series of ancestral variants; Yao et al., 2002) in human diseases is always a hot topic. The mtDNA encodes 13 peptides, 2 ribosomal RNAs and 22 transfer RNAs (Anderson et al., 1981). Recent studies showed that that mtDNA also encodes short mitochondrial peptides, such as Humanin, which plays an important regulatory role in cell metabolism and related diseases (Kal et al., 2024; Logan, 2017). Genetic variants in these mitochondrial peptides, such as m.A2672G (Secher et al., 2014), are also characteristic of mtDNA haplogroup(s). This means that the people in haplogroup U6a7a1a have Humanin S14G, a form of the peptide that is considered to be much more potent than normal Humanin (Li et al., 2013), would be a result of specific adaptation or selection. Disappointingly, there has not been any progress in determining if being in haplogroup U6a7a1a does indeed have benefits, but it continues to be an interesting possibility.

The recently published paper by Miller et al. (2024) has suggested that another variant m.C2639T in the Humanin peptide may also be beneficial. This variant leads to Humanin P3S and defining mutation haplogroup N1b. The researchers suggest this may be associated with increased longevity and a lessening of the pathological features seen in Alzheimer's disease (AD). At the present time, variant m.C2639T is only found in people who are in haplogroup N1b—a Eurasian haplogroup of about 10 million people based on its global distribution frequency. Interestingly, around 500,000 people in haplogroup N1b are Ashkenazi Jews, mostly belonging to a distinctive subgroup, N1b1b1, with a few also to be found in in the subgroup N1b1a2j1a (Brook, 2022). The proportions of haplogroups N, N1b and N1b1b1 are shown in Figure 1.

**FIGURE 1:**
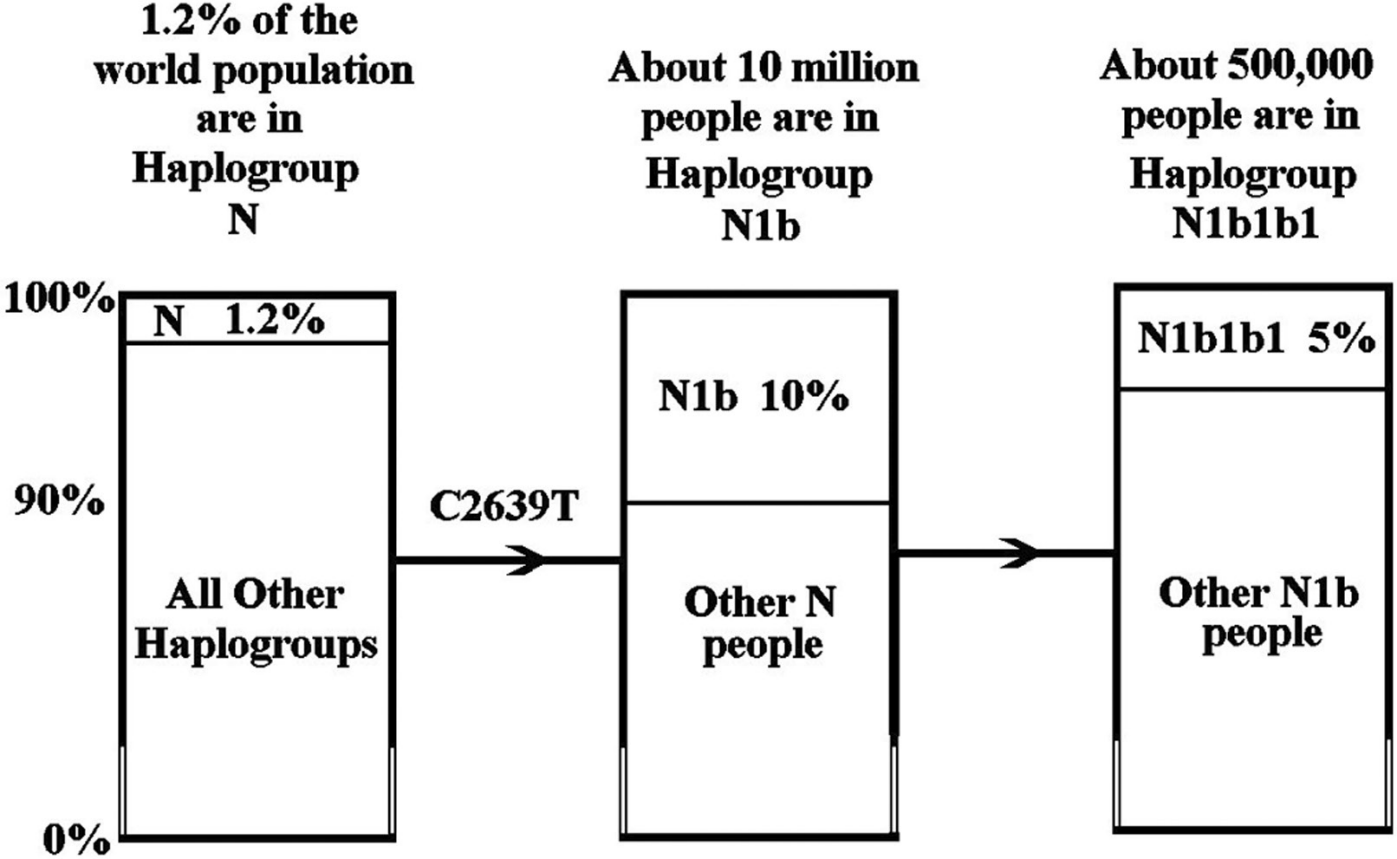
There are about 100 million people in haplogroup N and 10 million of them have the mutations C2639T and are therefore in haplogroup N1b. There are about 500,000 people in haplogroup N1b1b1and they are predominantly Ashkenazi.

Miller et al. (2024) explored the paradox that people carrying the allele APOE4 (Liu et al., 2013) have an increase in their risk of developing AD and decreased life expectancy, ‘nonetheless, some APOE4 carriers exhibit resistance to AD even in advanced age’. They looked to resolve the dilemma by studying a group of 146 women, who are ‘95 years of age or older and were principally of Ashkenazi genetic ancestry’. Samples were taken and analysed to obtain the complete mitochondrial DNA sequence and APOE4 status for each of the subjects. The results given in this paper, in respect of the centenarians in the study, can be summed up by saying, 6 subjects out of the 20 who had the APOE4 allele also had Humanin P3S, that is, an occurrence frequency of 30%; whereas 9 out of the 126 who did not have the APOE4 allele also had Humanin P3S, that is, frequency of 7%. This difference between these two groups suggests that being in haplogroup N1b is beneficial and increases the chances of living to be a centenarian—possibly because of the variant m.C2639T. The researchers also tested the use of synthetic Humanin P3S in an AD mouse model, and showed that the histological features of AD are lessened by this mutated form of Humanin, as compared to that of the non‐mutated peptide, but there was no data to show a beneficial effect of Humanin P3S on cognitive impairment.

Although the findings in the study by Miller et al. (2024) await further validation, this study raises as many questions as it answers: Does this effect refer to Humanin P3S or haplogroup N1b? What is the detailed mechanism underpinning this ameliorating effect? What is the structural basis for the short mitochondrial peptide to have such a striking effect? Are there any other variants in Humanin with similar or even better effect? The suggestion that Humanin P3S lessens the pathology seen in AD is promising and this might lead to the therapeutic use of Humanin P3S as a way of delaying the disease. As for the people who have the variant m.C2639T, it may be that they have an inheritance that protects them against the harmful effects of the APOE4 allele and increases their life expectancy. However, are there any trade‐off effects for this protection by Humanin P3S in counteracting with the deleterious effect of the APOE4 allele? Evidently, answers to these questions will broaden our knowledge about the role of mtDNA variants and different mitochondrial haplogroup in aging and disease.

## FUNDING INFORMATION

No funding information provided.

## CONFLICT OF INTEREST STATEMENT

None declared.

## Data Availability

None declared.
